# Genetics, diagnosis and management of colorectal cancer (Review)

**DOI:** 10.3892/or.2015.4108

**Published:** 2015-07-03

**Authors:** MARINA DE ROSA, UGO PACE, DANIELA REGA, VALERIA COSTABILE, FRANCESCA DURATURO, PAOLA IZZO, PAOLO DELRIO

**Affiliations:** 1 Department of Molecular Medicine and Medical biotechnology, University of Naples 'Federico II', I-80131 Naples; 2 Colorectal Surgical Oncology-Abdominal Oncology Department, Istituto Nazionale per lo Studio e la Cura dei Tumori, 'Fondazione Giovanni Pascale' IRCCS, I-80131 Naples; 3 Ceinge Biotecnologie Avanzate, I-80145 Naples, Italy

**Keywords:** colorectal cancer, genetic heterogeneity, molecular signaling pathways, epithelial-to-mesenchymal transition, hereditary colorectal cancers, early diagnosis, personalised care, minimally invasive surgery, conventional laparoscopic surgery, robotic surgery

## Abstract

Colorectal cancer (CRC) is the third most common type of cancer worldwide and a leading cause of cancer death. Surgery represents the mainstay of treatment in early cases but often patients are primarily diagnosed in an advanced stage of disease and sometimes also distant metastases are present. Neoadjuvant therapy is therefore needed but drug resistance may influence response and concur to recurrent disease. At molecular level, it is a very heterogeneous group of diseases with about 30% of hereditary or familial cases. During colorectal adenocarcinomas development, epithelial cells from gastrointestinal trait acquire sequential genetic and epigenetic mutations in specific oncogenes and/or tumour suppressor genes, causing CRC onset, progression and metastasis. Molecular characterization of cancer associated mutations gives valuable information about disease prognosis and response to the therapy. Very early diagnosis and personalized care, as well as a better knowledge of molecular basis of its onset and progression, are therefore crucial to obtain a cure of CRC. In this review, we describe updated genetics, current diagnosis and management of CRC pointing out the extreme need for a multidisciplinary approach to achieve the best results in patient outcomes.

## 1. Molecular basis of CRC

During colorectal adenocarcinoma development, epithelial cells from gastrointestinal trait acquire sequential genetic and epigenetic mutations in specific oncogenes and/or tumour suppressor genes, conferring them a selective advantage on proliferation and self-renewal (1,2). So, normal epithelium becomes hyperproliferative mucosa and subsequently gives rise to a benign adenoma that evolves into carcinoma and metastasis in about 10 years (3).

Sporadic colorectal cancers (CRC), due to somatic mutations, account for about 70% of all CRCs. Familial CRC, a group of diseases in which patients do not present a Mendelian inheritance, but only a familial predisposition to develop cancer, are about 10–30%, whereas hereditary diseases are about 5–7% (4). Germline minor variant and/or single-nucleotide polymorphisms (SNPs) in oncogene or tumour suppressor genes are responsible for the familial types of the disease, while inactivating mutations in the same genes cause hereditary CRC (5,6). The main hereditary CRC syndromes are hereditary nonpolyposis colorectal cancer (HNPCC) and adenomatous polyposis syndrome (7).

Normal gastrointestinal epithelium is organized along a crypt-villus axis. A pool of colon stem and progenitor cells, the most undifferentiated cell types that are able of self-renewal and pluripotency, are located at the bottom of the crypt. These cells migrate along the crypt-villus axis, simultaneously differentiating in all epithelial colon lineages, such as Paneth, goblet, enterocytes and enteroendocrine cells. In about 14 days they arrive at the top of the villus and undergo programmed cell dead (apoptosis) (8,9). This process is orchestrated from gradients of proteins, such as Wnt, BMP and TGF-β, together with extracellular matrix and stromal cells, that form the cell niche (10).

At the molecular level, CRCs are a very heterogeneous group of diseases. Loss of genomic integrity facilitates the accumulation of multiple mutations during the development of CRCs. Chromosomal instability (CIN), microsatellite instability (Min), aberrant DNA methylation and DNA repair defects are all mechanisms involved in colorectal epithelial cell transformation and all play a significant role in CRCs (11–14).

Several altered molecular signaling pathways are involved in CRC onset, such as Wnt/APC/β-catenin, phosphoinositide 3-kinase (PI3K)/AKT/glycogen synthase kinase-3β (GSK-3β), transforming growth factor-β (TGF)-β/Smad, NF- κb or mismatch repair genes (MMR). These alterations confer individual susceptibility to cancer, and are responsible for responsiveness or resistance to anti-tumor agents (15,16).

### Wnt/β-catenin pathway

It is the most frequently dysfunctional signaling in sporadic CRC. When Wnt ligand, a secreted glycoprotein, binds to its Frizzled (Fz) receptors, the multifunctional kinase GSK-3β is inactivated and β-catenin, that acts both as E-cadherin cell-cell adhesion protein and as a transcriptional activator, is stabilized, accumulated in the cytoplasm and finally translocated into the nucleus where it interacts with members of the lymphoid enhancer factor (LEF)/t-cell factor (TCF) and activates specific target genes. In the absence of Wnt-signal, casein kinase 1 (CK1) and the APC/Axin/GSK-3β-complex, target β-catenin for ubiquitination and proteasomal degradation by its phosphorylation, so preventing its nuclear translocation (17,19).

Wnt signaling contributes to tumour cell proliferation and inhibition of differentiation but it is also a critical mediator of endothelial function (20,21). Norrin, a non-Wnt ligand, binds selectively to Fz receptor subtype-4 (Fz4) and induces canonical Wnt signaling in conjunction with cell surface co-receptor LRP5 (9,10). Recently it has been demonstrate that Norrin is produced by human CRC, that it directly regulates endothelial cell proliferation and behavior, and that all of the critical components necessary to respond to Norrin signals are expressed by endothelial cells in the tumour microenvironment (22).

### PI3K/AKT pathway

PI3K/AKT/PTEN pathway, often found dysfunctional in same sporadic and hereditary CRC, activates cell growth and inhibit apoptosis in response to several extracellular stimulations, such as growth factors, cytokines, hormones, heat and oxidative stress, hypoxia and hypoglycemia.

Binding of growth factor to its receptor induces self-phosphorylation and activation of receptor itself. Consequently, PI3K is recruited at the plasma membrane level and so activated. Activated PI3K converts phosphatidylinositol (4,5)-bisphosphate (PIP2) into phosphatidylinositol (3,4,5)-trisphosphate (PIP3) and binding of PIP3 to AKT anchors it to the membrane allowing its phosphorylation and activation via phosphoinositide-dependent kinase-1 (PDK1) (23–25).

AKT acts in many cellular processes such as control of metabolism, translation, apoptosis and the cell cycle, by phosphorylating several target proteins, such as BAD (BCL-2 antagonist of cell death), caspase-9, mTOR (mammalian target of rapamycin), GSK3 and β-catenin (26–28). PTEN, a negative regulator of PI3K/AKT pathway, is a tumour suppressor gene, whose alterations are involved in several sporadic and hereditary CRC, that carries out its rule by dephosphorylating and downregulating PIP3 levels (29). Somatic mutations in this gene, such as small quantitative alterations of its expression, were found to be associated to different types of cancer (30). Furthermore, its germ-line mutations cause PTEN hamartoma tumors (PHTS) syndrome, a hereditary disorder predisposing patients to onset of multiple neoplasms (31–33). Recently it has been suggested that PTEN protein, as many others tumour suppressor genes, acts in a quantitative manner (34), and quantitative changes in its expression levels could have a role in phenotypic variability that PHTS patients have shown (35).

### Ras/Raf pathway

Ras/Raf signaling determines mitogen-activated protein kinase (MAPK) activation, a group of serine/threonine kinase proteins that mediate signal transduction from plasma membrane to the nucleus, in response to several extracellular stimulations, such as growth and mitogenic factors (36,37).

Ras genes (H, K and N) are localized on chromosome 12 and encode for small proteins with GTPase activity bound to the plasma membrane. Ras mutations usually activate Ras signaling by enhancing GTP levels and so mediating Raf proteins (A, b and c) phosphorylation and activation (38). Raf proteins, in turn, transduce signaling between MEK (1 and 2) and ERK (1 and 2) proteins, inducing transcription of genes involved in the cell cycle, and transcription regulation, such as Myc, cyclin-D/CDK (39,40).

### NF-κB pathway

NF-κB is a signaling pathway that takes part in cell proliferation and inflammation mechanisms. It consists of five subunits acting as transcription factors, RelA/p65, c-Rel, RelB, p50/NF-κB1 and p52/NF-κB2, that are able to dimerize and are sequestered in the cytoplasm by Iκb proteins. The IKK complex, consisting of two catalytic (IKKα and IKKβ) and one regulatory (IKKγ) subunits, represents the major regulator of this pathway. It acts by phosphorylating Iκb proteins targeting them for proteasomal degradation. Iκb degradation releases NF-κB proteins in the cytoplasm. Thus, they are free to translocate into the nucleus and activate transcription of specific genes (41,42).

### GSK-3β: a central regulator in cross-talk between the CRC pathways

All the molecular pathways cross-talk with each other and are regulated by one another. Interestingly, concerted regulation exists between NF-κB, Wnt and other adhesion proteins such as E-cadherin. E-cadherin directly binds to β-catenin and NF-κB sequestering them at the plasma membrane level and subtracting them from the nucleus. When the transcription of WNT5A is activated by NF-κB, WNT5A binds its specific Wnt receptor inducing transcription of target genes such as snail, CD44 and YAP1. Snail upregulation, in turn, represses E-cadherin expression releasing β-catenin and NF-κB, available to translocate into the nucleus (43,44).

GSK-3β is a protein that has a pivotal role in cross-talk between Wnt and NF-κB (45). In colon and pancreatic cancer cells, GSK-3β positively regulates NF-κB activity and its activation confers a selective growth advantage to these cells, therefore acting as a tumour promoter (46). The molecular mechanisms underlying GSK-3β/NF-κB interaction remain to be further investigated. More than 100 proteins, involved in a wide spectrum of cellular processes, are substrates of GSK-3β, of which β-catenin and NF-κB inhibitor iκb are the most well known. Genes upregulated by β-catenin/TCF/LEF and/or NF-κB include proto-oncogenes, such as c-Myc and cyclin-D1, and genes regulating cell invasion/migration, such as Snail, CD44 and MMP-7 (47).

### Epithelial-to-mesenchymal transition (EMT): a common mechanism in molecular heterogeneity

Recently, it was suggested that EMT could be a common biological mechanism in cancers, representing a good target for therapeutic intervention. EMT consists in an essential phenotypic conversion of epithelial cells into cells with mesenchymal phenotype. It is a reversible process that often occurs during embryonic development and tissue remodeling and also plays a critical role in early event occurring in invasion and metastasis of many types of cancer, including CRC (48). EMT regulation is orchestrated by a group of transcription factors, including snail, slug, zeb1 and twist, but tumour microenvironment is also involved in this conversion through different signals, such as TGF-β, EGF, Wnt and Notch (49,50).

During EMT, epithelial cells lose E-cadherin expression, that specifically guarantees the epithelial phenotype, destroy their intercellular adhesion, acquire mesenchymal characteristics and increase migratory and invasive properties (51). Furthermore, EMt program induces stem cell specific gene expression, thus promoting self-renewal capability (52,53). Thus, understanding the specific molecular mechanisms sustaining EMT is of great relevance to block cancer progression.

TGF-β pathway represents the main signaling that regulates EMT. Binding of TGF-β to its receptor II (TGF-β-RII) promotes RI receptor recruitment on the complex at the membrane level. The activated complex induces Smad proteins phosphorylation, that, in turn, translocates into the nucleus, switching on transcription of specific genes, such as Snail, Slug, Twist and ZEB, all genes that are involved in EMT (54). We recently demonstrated that LiCl, a GSK-3β inhibitor, induces mesenchymal-to-epithelial transition (MET) *in vitro*, suggesting that LiCl and GSK-3β could represent, respectively, interesting drug and target for CRC therapy (55).

## 2. Hereditary CRC and molecular diagnosis

Hereditary CRCs represent about 7–10% of CRC and include HNPCC, adenomatous (FAP and MAP) and hamartomatous (PJS, JPS, PHTS) polyposis syndromes (56–58). The genes whose alteration are involved in their onset are now well known (Table I).

Differential diagnosis is essential for the management and cancer prevention of the affected individuals, because of each syndrome has its own distinctive organ-specific manifestation and each requires a different surveillance strategy.

Patients with germline causative CRC mutations have a high lifetime risk of gastrointestinal and extra-intestinal carcinoma and their first-degree relatives have a high risk of recurrence of the syndrome. Characterization of a causative mutation in leukocyte DNA is essential for the differential diagnosis among the various hereditary CRC syndromes, assessment of the risk of recurrence (autosomal dominant versus autosomal recessive inheritance), determination of familial cancer risks based on gene-specific cancer associations and predictive testing of asymptomatic at risk individuals.

The role of the molecular genetic findings in treatment decisions, on the other hand, is limited because identification of a germline mutation rarely allows any estimation of the likely course of the disease. According to the international literature data, we suggest that next-generation sequencing (NGS) is today the best and the most efficient technique for molecular diagnosis of hereditary colorectal polyposis syndrome, and hereditary/familial CRC. Moreover, even if no mutation is found, the patient with hereditary cancer syndromes still needs to be treated appropriately and clinical follow up should be initiated even before mutation testing is complete (59).

## 3. CRC diagnosis

### CRC clinical manifestations

Clinical manifestations of CRC depend on the location of the lesion. Both right and left colon lesions occasionally cause hematochezia, but more often bleeding is occult, causing anemia and fatigue. Rectal lesions cause hematochezia, bleeding and tenesmus. Up to 30% of patients with colorectal carcinoma are primarily diagnosed in an acute stage with sub/obstructing symptoms (60,61). In 20–25% of patients with colonic cancer and in 18% of patients with rectal cancer, metastases are present at the time of the first diagnosis (62,63). Although liver represents the most common metastases localization in CRC patients, 2.1% of these patients show lung metastases (64), with frequency about three times higher for patients with rectal cancer than for patients with colon cancer.

### Clinical diagnosis and staging

Appropriate diagnosis and staging are crucial to ensure a correct treatment strategy. In the last 10 years the mortality rate of CRC has decreased by more than 20% due to the rising developments in diagnostic techniques and optimization of surgical, adjuvant and also palliative therapies (62). A complete colonoscopy up to the cecum, coupled with biopsy for histopathological examination, is considered the gold standard to diagnose colorectal lesions, in view of its high diagnostic performance (65,66). This procedure allows the tumour localization and possibly the endoscopic excision of polyps, so simultaneously representing a diagnostic and a therapeutic opportunity (67).

The best results are exhibited for lesions >6 mm, showing sensitivity and specificity of about 98 and 99%, respectively (68). However, a substantial proportion of patients will have an incomplete colonoscopy due to poor bowel preparation, poor patient tolerance, obstruction or other technical difficulties. In these cases, additional computed tomography-colonography (CT or CTC) can contribute to the CRC diagnosis (69,70), as a potential alternative to the endoscopy, especially in patients with stenotic tumour and/or when colonoscopy turn out to be incomplete or difficult. Furthermore, ct has been established as a highly sensitive and specific diagnostic modality for lesions >10 mm (71).

Despite such promising data, CTC does not offer the opportunity of taking biopsies or immediate polypectomy and the patient needs to return for a colonoscopy, in case of detected lesions. Tumours with distal extension to 15 cm (as measured by colonoscopy) from the anal margin are classified as rectal, while more proximal tumours are classified as colonic. Rectal digital examination can identify cancers up to 8 cm above the dentate line. Imaging plays a crucial role in the diagnosis, staging assessment for specific (e.g., neoadjuvant) therapy and follow up of patients with colon and rectal cancer with the main function of defining the locoregional extent, identifying synchronous lesions and distant metastases.

CRCs are classified according to local invasion depth (T stage), lymph node involvement (N stage) and presence of distant metastases (M stage) (72). These stages are combined into an overall stage definition, which provides the basis for therapeutic decisions. Specifically, classification according to tumour, node and metastases (TNM) and Union Internationale Contre le Cancer (UICC) stage offers valuable prognostic information and guides therapy decisions.

The most used imaging modalities for staging of CRC are chest/abdomen/pelvis CT (63,73,74) and magnetic resonance imaging (MRI). CT has a sensitivity of 74–84% and a specificity of 95–96% in detection of CRC liver metastases (64,74). MRI evaluates liver lesions <1 cm in size with a sensitivity of 80–88% and a specificity of 93–97% (75). However, ct has shown to be poor in identifying nodal disease (76,77). Its specificity for lymph node staging is 55%, while the sensitivity is 76% (64). Nodal size is too unreliable as a predictor for malignancy and should not be used as an absolute tool to define whether lymph nodes are involved or not. In preoperative imaging of rectal cancer, MRI is recommend for local staging (78,79) and whole body CT for detection of distant metastases (80–82).

MRI is able to accurately define the local extension of rectal tumour, the mesorectal fascia involvement, circumferential resection margin (CRM) (78) and the relationship of the tumour to the sphincter complex. It has a high accuracy in detecting extramural spread and in identifying tumours with good (<5 mm invasion) vs. poor (>5 mm invasion) prognostic features. Moreover, MRI represents the most accurate imaging tool for identifying nodal disease with a sensitivity of 85% and specificity of 97%, not by a size criteria but better with a definition of border irregularity and heterogeneity. MRI therefore is essential to accurately stage patients with rectal cancer in order to select the indication to a preoperative treatment or defining the extent of radical surgery.

Endorectal ultrasound (ERUS) is another well established modality for the evaluation of the integrity of the rectal wall layers. With accuracies for T staging ranging between 69 and 97%, endorectal ultrasonography is currently the most appropriate imaging modality for the assessment of T1 tumours. It may also detect regional adenopathy, with a lower accuracy to identify nodal involvement compared to both CT and MRI. As described by swartling *et al* (83), the combination of MRI with ultrasound improves diagnostic accuracy.

## 4. Management of colon cancer

Primary colon cancers without systemic disease are treated mainly by surgery with complete mesocolic excision (CME) (84,85) with arteries and veins ligated as close as possible to the main vascular trunk to have lower local recurrence rate and improved survival (86,87). The concept of CME is similar to the total mesorectal excision (TME) for rectal cancer and allows an excellent oncological outcome with a 5-year cancer specific survival rate of 91.4% in stage ii, and 70.2% in stage III CC (87). Colonic segmental resection is performed according to the site of the tumour; right hemicolectomy transverse colectomy, left hemicolectomy or total colectomy are the most common surgical procedures and it is always indicated in absence of metastases.

In the emergency setting, when presenting symptoms of obstruction, perforation and bleeding, segmental colectomy for resection of the tumour, with or without fecal diversion, is indicated. In elderly patients with acute malignant colonic obstruction in whom emergency surgery carries high risks of morbidity and mortality, in presence of unresectable metastatic lesions or as initially palliation of obstructing CRC, the use of self-expandable metal stents (SEMS) is gaining wide acceptance, also to allow a quick start of neoadjuvant chemotherapy or chemoradiation. The colonic stent insertion effectively decompresses the obstructed colon and surgery can be performed electively at a later stage avoiding a derivative ostomy, whenever possible (88–90).

Minimally invasive surgery is a safe and valid alternative to open surgery in the treatment of colon cancer with less postoperative pain, shorter duration of ileus and hospital stay. Also, randomized trials have demonstrated similar oncologic outcomes compared with open surgery. Conventional laparoscopic surgery (CLS) with CME, is today considered a safe technique and spread all over the world; single-incision laparoscopic surgery (SILS) appears to offer cosmetic advantages over CLS, with no compromise of surgical morbidity, oncological appropriateness or increased cost. Recent studies show that colorectal resections for cancer with SILS have short-term results comparable with CLS (91–95). Laparoscopic and robotic surgery for colon surgery have the same advantages in terms of faster recovery, but robotic-assisted colectomy significantly increases the costs of care without providing clear reductions in overall morbidity or length of stay, so a critical appraisal of the benefits offered in comparison with the resources consumed is undergoing (96,97).

## 5. Management of rectal cancer

Rectal cancers can be divided into 4 groups: very early (some cT1), early (cT1–2, some cT3), intermediate (most cT3, some cT4) and locally advanced (some cT3, most cT4) but, for cancer staging we have to consider other important factors as distance from the anal verge, circumferential margin (crm) (98), nodal (cN)-stage, vascular and nerve invasion. Rectal cancer has a distal extension to 15 cm or less from the anal margin.

### Treatment of very early rectal cancer

For the very early tumours (stage 1) and for the malignant polyps [Haggitt 1–3, T1 sm 1(−2?) N0], after adequate staging by ERUS, rectal MRI and CT scan, local excision could be considered, by means of traditional transanal procedure or by a video-assisted technique, both transanal endoscopic microsurgery (TEM) (99,100) or transanal minimally invasive surgery (TAMIS) (101,102).

### Treatment of early rectal cancer

Surgery is the mainstay of treatment for early rectal tumours: TME is the appropriate procedure, both performed laparoscopically or open. Minimally invasive approach is slow to become diffusely utilized due to the long learning curve of laparoscopic surgery, when compared to open surgery it has shown better short-term clinical outcomes and some evidence of comparable oncologic results are now arising. Several trials have compared the different surgical techniques in terms of oncological radicality and survival outcome.

The CLASICC trial comparing laparoscopic to open surgery and including rectal resections, showed an increased positivity of the CRM in the laparoscopic group, but no significant differences in terms of survival rates after 6 years of follow-up when compared to open surgery (103,104).

The COREAN trial (105) compared laparoscopy to open surgery after neoadjuvant chemoradiotherapy (CRT), showed no significant differences in terms of CRM positivity between 2 groups: 4.1% in the open group and 2.9% in the laparoscopic group.

The COLOR II study (106) showed a higher CRM positivity in the open surgery compared to laparoscopic group (22 vs. 9%, P=0.014), but no significant differences in terms of morbidity, mortality, and complication rates.

More studies are therefore needed to verify the superiority of one surgical technique over the others in terms primarily of oncological radicality and disease free survival.

Robotic surgery, finally, would seem to overcome the technical difficulties of laparoscopy, but there are still no reliable data demonstrating its superiority in terms of oncological results compared to open and laparoscopic surgery (29,30,107,108).

Robotic approach resulted in a lower percentage of conversion to open surgery (109,110) and a superiority of robotic rectal resection in recovery of urinary voiding and sexual function (111–113). Data from two multicentric studies comparing robotic to laparoscopic TME (ROLARR and COLRAR) are ongoing and results are awaited to better define the role of robotic rectal surgery (114).

### Treatment of intermediate and locally advanced rectal cancer

Neoadjuvant therapy in [(C)RT] allows radical surgery with TME also in locally advanced rectal cancer. This approach has led to a reduction of recurrence rates from 30–40% of a few decades ago down to 5–10% or even lower figures. Adjuvant and neoadjuvant approach to locally advanced rectal cancer has been long debated: while a NIH Consensus Conference in the early 1990s stated that the most appropriate treatment was postoperative CRT (115,116), in Europe numerous trials supported RT before surgery with moderately high and never lower than 30 Gy doses. This approach has also led to a decrease of the local recurrence rate (117).

Neoadjuvant chemoradiation became standard practice after the publication of the results of the German trial (118). The choice between standard long course chemoradiation and short course radiation is still related to the 'tradition' of the group.

Several studies were performed, many are still in progress and many of them showed that both options have to be considered valid (118,119).

The stockholm III trial (120,121) regarding the fractionation of radiotherapy (RT) and timing of surgery for rectal cancer, has randomized patients to preoperative short-course RT with two different intervals to surgery: 5×5 Gy and surgery within 1 week (group 1) or after 4–8 weeks (group 2) or long-course RT 25×2 Gy and surgery after 4–8 weeks (group 3). These two different preoperative RT regimens has shown that patients of group 1 were associated with more postoperative complications than the other and that the experimental schedule (group 2) was shown to be feasible and as safe as the established treatments. However, results about local recurrences, will be available later in 2015.

Bujko *et al* (122) published long-term results of a randomized trial comparing preoperative short-course RT with preoperative conventionally fractionated chemoradiation for rectal cancer. Chemoradiation (50.4 Gy in 28 fractions of 1.8 gy, bolus 5-fluorouracil and leucovorin) and surgery 4–6 weeks later did not increase survival, local control or late toxicity compared with short-course RT alone.

Ngan *et al* (123) published the results of a randomized trial comparing short RT and CRT long-course having as end point the local recurrence in T3 rectal cancer. Two different treatments were offered: in one, patients underwent pelvic RT 5×5 Gy in 1 week, early surgery and six courses of adjuvant chemotherapy; in the other, long course RT 50.4 Gy, 1.8 Gy/fraction, in 5.5 weeks, with continuous infusional fluorouracil 225 mg/m^2^ per day, surgery in 4–6 weeks, and four courses of chemotherapy were given. The final results showed that there were not significant differences between the two schedules in terms of local recurrence, except that long RT was more effective in reducing local recurrence only for distal tumours.

Siegel *et al* (124) published a randomised study comparing short RT plus TME-surgery within 5 days in the first group and long RT with continuous infusion 5-fluorouracil plus TME-surgery 4–6 weeks later in the second group. After surgery all patients had adjuvant chemotherapy for 12 weeks. No difference was shown in terms of local recurrence rate between the two groups.

### The choice of chemotherapy in neoadjuvant treatment in locally advanced rectal cancer

5-fluorouracil (5-FU) in infusion is now the drug commonly used in neoadjuvant treatment. The oral capecitabine gives the same effects and is much easier to manage by both the oncologist and patient (125).

Combinations of 5-FU and other cytotoxic drugs such as oxaliplatin and irinotecan, and targeted drugs, have been extensively explored during the past decade. Multiple phase II studies in the so-called 'locally advanced rectal cancer' have claimed better results [more down-sizing, higher pathological complete rates (pCR)]. When cetuximab was added to CRT with capecitabine and neoadjuvant chemotherapy with capecitabine-oxaliplatin in a randomized phase II study, the primary endpoint, pCR rate, was not increased, but more radiological responses (89 vs. 72%, P=0.002) and improved OS (96 vs. 81% at 3 years, P=0.04) were seen in the KRAS wild-type population (n=90) (126).

## 6. Towards personalized care

CRC is the third leading cause of cancer mortality worldwide and prognosis for CRC patients is directly related to the timing of diagnosis. When detected early, it is often cured with surgery alone. For more advanced or metastatic disease, chemotherapy is added to surgical treatment. As described so far, several alterations at molecular level favour CRC onset, progression and metastasis. Molecular characterization of cancer associated mutations gives valuable information on disease prognosis and response to therapy.

Current clinical methods for prognostication in CRC are based on the American Joint committee on cancer (AJCC), TNM staging classification. In the real life the relationship between TNM stage and prognosis is much more complex, because each cancer stage is also a heterogeneous group (127).

The advent of NGS technology allowed a better, rapidly and also cheaper molecular characterization of cancers. However, clinically relevant biomarkers can be measured in a variety of ways, from mutations in the coding DNA to dysfunctional proteins found by specific activity assays (128,129).

Despite significant progress in colon cancer research, the translation of genetic discoveries into diagnostic tests for colon cancer patients has been difficult and the function of most of the mutations remain ambiguous. Few are genetic bio-markers that today start to have a clinical value as prognostic and/or therapeutic predictive markers, including MSI status and EGF signaling pathway.

There are now strong evidence that MSI is associated with improved prognosis but decreased response to 5-FU-based chemotherapy (130–133).

Concerning EGF signaling, two monoclonal antibodies against EGFR, cetuximab and panitumumab, are now commercially available (134). They are approved for use in combination with 5-FU, leucovorin and oxaliplatin (FOLFOX) or 5-FU, leucovorin and irinotecan (FOLFIRI) for stage IV metastatic CRC (135–137). Unfortunately, efficacy of these regimens remains modest with 8–25% objective response rates because of great Ras/Raf mutation rate that are present in CRC (138). It has been suggested that PTEN alterations and PI3KCA mutations also give the same effect. With implementation of NGS towards personalized care, molecular characterization of sporadic colorectal cancers is assuming increased importance. Kras, Braf and MSI are already routinely used in clinical practice, however, in our opinion, PTEN and/or PI3KCA determination needs to be improved as diagnostic, prognostic and predictive molecular biomarker in CRC management. We also suggest that GSK-3β could represent an interesting target for colorectal cancer therapy, in view of its central role in molecular signaling pathway cross-talk and EMT regulation.

## 7. Conclusion

As described in this review, CRCS represent a very heterogeneous group of disorders both in the biological behavior and at molecular level, in which different patterns of mutations contribute to the onset and progression and are responsible for specific aggressiveness and also for response to the therapy. NGS technology are going to improve the molecular characterization of sporadic and hereditary diseases allowing standardization of personalized care. Therefore, it is becoming of great importance to standardize the methodology for new predictive, diagnostic and prognostic biomarkers in colorectal cancer, such as PTEN and/or PI3KCA. We also believe that discovery of new therapeutic targets represents the goal of biomedical research on CRC at the moment, since majority of CRC are insensitive to EGFR inhibitor therapy, because of their positivity for mutations in the genes Kras, Braf, PI3KCA and PTEN. We suggest that epithelial to mesenchymal transition could represent an interesting mechanism for therapeutic target, because it seems a common mechanism on extensive molecular heterogeneity that characterises CRCs. Moreover, as we recently suggested, GSK-3β could represent an interesting target for colorectal cancer therapy (55).

## Figures and Tables

**Table I:** Genes involved in hereditary colorectal cancer syndrome.

Syndrome	Gene	Hereditary
Hereditary non-polyposis colorectal cancer (HNPCC)	MLH1, MSH2, MSH6, MLH3, MSH3 and PMS2	Dominant
Turcot syndrome (TS)	MMR or APC	Dominant or Recessive
Familial Adenomatous Polyposis (FAP)	APC gene	Dominant
MUTYH-associated polyposis (MAP)	MUTYH	Recessive
Peutz-Jeghers syndrome (PJS)	STK11/LKB1	Dominant
PTEN hamartoma tumors syndrome (PHTS)	PTEN	Dominant
Juvenile polyposis syndrome (JPS)	SMAD4-BMPR1A	Dominant
Polymerase Proofreading-Associated	POLD1-POLE	Dominant
Polyposis (PPAP)		

## Abbreviations

CRC: colorectal cancer
SNP: single-nucleotide polymorphism
CIN: chromosomal instability
Min: microsatellite instability
EMT: epithelial-to-mesenchymal transition
CTC: computed tomography-colonography
CT: computed tomography
MRI: magnetic resonance imaging
CRM: circumferential resection margin
ERUS: endorectal ultrasound
CME: complete mesocolic excision
SEMS: self-expandable metal stents
CLS: conventional laparoscopic surgery
SILS: single-incision laparoscopic surgery
crm: circumferential margin
TEM: transanal endoscopic microsurgery
TAMIS: transanal minimally invasive surgery
TME: total or partial mesorectal excision
CRT: chemoradiotherapy
RT: radiotherapy
pCR: pathological complete response rate
TNM: tumour, node and metastasis
NGS: next-generation sequencing
